# Cross-cultural adaptation of the Neonatal Medical Index (NMI) to Brazil

**DOI:** 10.1590/1984-0462/2024/42/2023164

**Published:** 2024-04-29

**Authors:** Marcelo Dias, Silvana Alves Pereira, Roberta Costa, Sérgio Tadeu Martins Marba, Dayane Montemezzo

**Affiliations:** a Fundação Catarinense de Educação Especial, São José, SC, Brazil.; b Universidade Federal do Rio Grande do Norte, Natal, RN, Brazil.; c Universidade Federal de Santa Catarina, Florianópolis, SC, Brazil.; d Faculdade de Ciências Médicas da Universidade Estadual de Campinas, Campinas, SP, Brazil.; e Universidade do Estado de Santa Catarina, Florianópolis, SC, Brazil.

**Keywords:** Premature infant, Childcare, Cross-cultural adaptation, Translation, Validation studies, Recém-nascido prematuro, Cuidado da criança, Adaptação transcultural, Tradução, Estudo de validação

## Abstract

**Objective:**

To perform a cross-cultural adaptation and assess the content validity of the Neonatal Medical Index (NMI) for the Brazilian context.

**Methods:**

The cross-cultural adaptation was completed in six steps, including translation, synthesis of translations, back translation, submission to an expert committee, testing of the prefinal version, and appraisal by the original author. The expert committee assessed the equivalence between versions based on the percentage of agreement, and content validity was evaluated using the content validity index (CVI) for each item of the scale (I-CVI) and for the overall scale (S-CVI) in terms of representativeness and clarity. Participants of the prefinal version also evaluated the CVI for clarity.

**Results:**

After two evaluation rounds of the expert committee it was attained 98% agreement, attesting to the equivalence between the instrument versions, maximum values for representativeness I-CVI and S-CVI/Ave (1.00), and high values for clarity I-CVI (all items ≥0.97) and S-CVI/Ave (0.98). The expert committee members defined that the Brazilian version of the instrument would be called *Índice Clínico Neonatal* (NMI-Br). The NMI-Br reached high values of CVI for clarity (all I-CVI ≥0.86 and S-CVI/Ave=0.99) among the participants of the prefinal version.

**Conclusions:**

The NMI-Br is the Brazilian version of the NMI, obtained in a rigorous cross-cultural validation process, counting with adequate values of content validity.

## RESUMO

**Objetivo:**

Realizar a adaptação transcultural e avaliar a validade de conteúdo do *Neonatal Medical Index* (NMI) para o contexto brasileiro.

**Métodos:**

A adaptação transcultural foi realizada em seis etapas, incluindo tradução, síntese das traduções, retrotradução, submissão a um comitê de especialistas, teste da versão pré-final e avaliação do autor original. O comitê de especialistas avaliou a equivalência entre as versões com base na porcentagem de concordância, e a validade de conteúdo foi avaliada por meio do índice de validade de conteúdo (IVC) para cada item da escala (I-IVC) e para a escala geral (S-IVC) em termos de representatividade e clareza. Os participantes da versão pré-final também avaliaram o IVC quanto à clareza.

**Resultados:**

Após duas rodadas de avaliação do comitê de especialistas obteve-se 98% de concordância, atestando a equivalência entre as versões do instrumento, valores máximos para representatividade I-IVC e S-IVC/Ave (1,00) e altos valores para clareza I-IVC (todos os itens ≥0,97) e S-IVC/Ave (0,98). Os membros do comitê de especialistas definiram que a versão brasileira do instrumento se chamaria Índice Clínico Neonatal (NMI-Br). O NMI-Br alcançou altos valores de IVC para clareza (todos I-IVC ≥0,86 e S-IVC/Ave=0,99) entre os participantes da versão pré-final.

**Conclusões:**

O NMI-Br é a versão brasileira do NMI, obtido em rigoroso processo de validação transcultural, contando com valores adequados de validade de conteúdo.

## INTRODUCTION

Preterm infants are commonly classified based on gestational age and birth weight,^1^ which are easy and practical to assess and report. However, relying solely on these parameters does not fully capture the complexity of the infant´s clinical history. For instance, two infants born at 29 weeks of gestational age and weighing 1400g can exhibit diverse clinical trajectories during their Neonatal Intensive Care Unit (NICU) stay, experiencing varied complications and outcomes.^2^ To address this limitation, a scoring system named Neonatal Medical Index (NMI) was developed to assess the severity of illness in infants during their NICU stay.^3^ This instrument provides a simple, objective, and easy-to-score process, utilizing data available in the NICU discharge report to summarize the preterm infant’s prior clinical course.^3^ The NMI was developed based on two fundamental principles: birth weight and the need/duration of mechanical ventilation. The classification involves two steps: first, based primarily on birth weight, and second, according to certain complications, ranges from I to V, with I representing preterm infants free of significant clinical problems and V characterizing infants with the most serious complications.^3^

One important characteristic of the NMI classification is its potential to predict later mental and motor development, particularly for infants born ≤1500g.^3,4^ This classification has been widely applied in various research scenarios, serving as a morbidity index to study infant development,^4,5,6,7^ investigate NICU routines,^8^ explore family support,^9^ and function as a screening tool for intervention programs.^10,11^

To our knowledge, no other indexes have been used as a morbidity index to classify premature infants. Existing instruments that predict the development of this population, such as the Bayley Scales of Infant Development^12^ and the Hammersmith Neonatal Neurological Examination,^13^ require infant evaluations conducted by trained professionals at specific ages, while the NMI is completed based on NICU discharge reports without subjecting the infant to further evaluations. It should be noted that the NMI does not replace a neurobehavioral examination, but it can serve as a rapid screening tool for identifying high-risk preterm infants who may require priority access to early developmental intervention programs.^10,11^

The NMI could prove to be a valuable instrument when applied in Brazil, a country where prematurity is a significant public health issue, with a high annual rate of premature births.^14,15^ Prematurity is listed among the main risk factors for children with special healthcare needs.^16^ In this context, the NMI may serve as an important tool for shaping public health policies aimed at monitoring infants discharged from the NICU and for infant follow-up services, acting as a criterion for referring the highest-risk infants to early stimulation services.

Considering that the NMI has not yet been translated and adapted for the Brazilian context, there was no decrease in the rate of sequelae (although there is a progressive improvement in survival in low- and middle-income countries),^17^ as well as the presence of long waiting lists for early intervention services indicated for all infants at risk of developmental delays,^18,19^ the objective of this study was to develop the cross-cultural adaptation and assess the content validity of the NMI for use in Brazil. The authors believe that providing a tool capable of predicting the risk of developmental delays at the time of hospital discharge can significantly aid in the early regulation of rehabilitation services.

## METHOD

This is a methodological study of cross-cultural adaptation (CCA)^20^ and content validity evaluation of the NMI, carried out from July 2021 to July 2022, approved by the Research Ethics Committee under CAAE No 47829521.3.0000.0118.

Prior to starting this study, Dr. David K. Stevenson, MD, Ph.D., Professor of Pediatrics at Stanford University School of Medicine (California, USA), who developed the NMI^3^, authorized its CCA. Dr. Stevenson has appointed Dr. Heidy Feldman, MD, Ph.D., Professor at Stanford University School of Medicine (California, USA), to accompany the CCA.

The NMI CCA was based on an international guideline which consists of six stages^20^ as described below:

Stage 1 - Initial translation: The original English NMI version was translated into Brazilian Portuguese by two independent bilingual translators, whose source language was Brazilian Portuguese. One of the translators was a nurse, aware of the NMI concepts, while the other, a *naïve* translator, had no medical background and was unaware of the concepts involved in NMI. This stage produced two translation versions (T1 and T2).

Stage 2 - Synthesis: The T1 and T2 were compared and synthesized in a new version (T3), containing the most appropriate translated terms defined by consensus between the translators, accompanied by the main researcher of this study. In addition, a written report was prepared, carefully documenting each of the issues addressed, and how they were resolved in the T3 version.

Stage 3 - Back translation: Two professional translators, whose source language was English and fluent in Brazilian Portuguese, both without a medical background, unaware of the original instrument and the purpose of the study, back translated the T3 from Portuguese to English. They worked independently and produced two independent back-translation versions (BT1 and BT2). There is no recommendation about a synthesis version of the back translations in the guideline used in this CCA, but it was prepared, informing which terms were translated differently.

Stage 4 - Expert committee (EC): The EC was developed in two rounds until the desired equivalence and agreement rates between the members were achieved.^20,21^ The EC members were chosen for convenience, aiming to include professionals from different regions of Brazil. The first round was composed of ten members: two neonatologists (one with master´s degree [MD] in health sciences and the other specialized in Neonatology), two nurses (one MD in health sciences and the other with MD in nursing), two physical therapists (one specialized in pediatrics and the other in neonatal intensive care), two speech therapists (both with MD in health sciences), all of them working in NICUs from 2 to 18 years (62% for <10 years, and 38% for >10 years), a university professor (physical therapist, Ph.D. in health sciences) expert in CCA, and also a premature infant’s mother (graduated in Business Administration, single mother of an only child born as an extremely preterm infant and that developed mental and motor handicap), representing the population^22^ evaluated by the instrument. These members lived in different regions of Brazil (50% South, 30% Southeast, 10% North, and 10% Northeast). The EC second round was composed of eleven members: nine of the EC’s first-round members (one of the neonatologists could not participate), plus two translators, one who participated in stage 1 (bachelor’s degree in nursing) and another who participated in stage 2 (professional translator with doctoral degree) of the CCA.

Each EC member working independently, received by electronic mail a set of documents containing (1) a cover letter asking for their participation, describing each step of the process and informing the conceptual underpinnings and measuring model being used,^21,22^ (2) all abovementioned NMI versions with comments about the translation process written in previous stages, and (3) the evaluation forms containing (A) dichotomous questions to assess semantic, idiomatic, cultural and operational equivalence to complete if they agree or not with the translated item and if necessary, they could suggest changes in the translation of the T3 version items, and (B) two 4-point Likert scales, one to assess relevance (1=the item is not relevant, 2=the item needs major revision to be relevant, 3=the item needs minor revision to be relevant, 4=the item is relevant)^22^ and another to assess clarity (1=not clear, 2=slightly unclear, 3=clear, 4=very clear) of each T3 item. The scoring of these measures was used to quantify the content validity.

Stage 5 - Test of prefinal version: Considering that the NMI must be understandable by the NICU health team, because they are responsible for collecting the necessary data to classify the NMI, the field test of the prefinal version was applied to 94 different health professionals, with experience in NICU, who accepted the invitation publicized by social media (WhatsApp®) to participate in this study. These professionals completed an electronic form (Google Forms®), evaluating the clarity of each NMI item, using a 4-point Likert scale (1=not clear, 2=slightly unclear, 3=clear, 4=very clear). Afterward, they evaluated the instructions, the items, and the scores of the prefinal version. Moreover, they could report their doubts and propose suggestions to clarify the instrument.

Stage 6 - Submission of documentation to the original author: Dr. Stevenson and Dr. Feldman were consulted at the end of the last three stages of the CCA. On these occasions, adaptations of the terms and the reasons for the changes were informed and authorization to proceed with the CCA was requested. At the final stage, they certified that a reasonable translation had been achieved.

**Figure 1:**
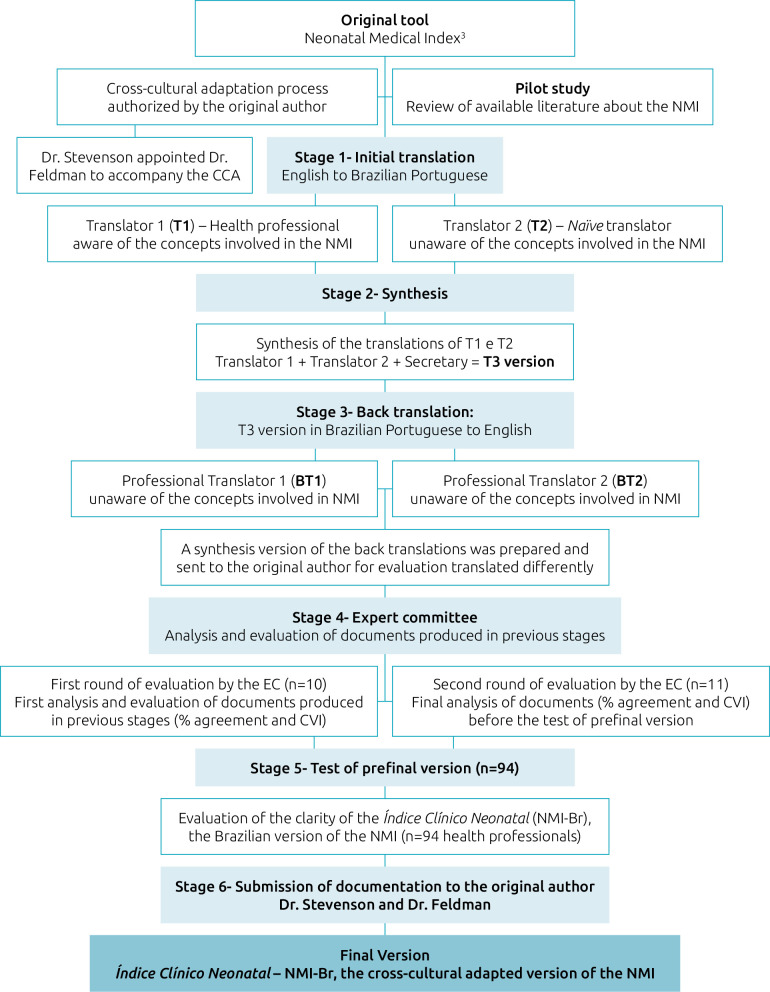
Flowchart of the cross-cultural adaptation process of the Neonatal Medical Index.

The stages of the NMI translation and CCA process are presented in Figure 1.

Two measures were assessed in stage 4 to analyze the data:

1. Percentage of agreement (% agreement) used to access semantic, idiomatic, conceptual and operational equivalence, and compatibility between the original and translated version) calculated by the formula % agreement=[(number of raters agreeing/total number of raters) x 100]^23^ and
2. Content validity index (CVI) used to assess the relevance and the clarity of the translated items. The CVI was calculated for each item individually (I-CVI=number of experts giving a rate of either 3 or 4/number of experts) and for the overall scale (S-CVI/Ave=average of the I-CVI for all items of the scale).^24^

The CVI was also used to assess the clarity of the instrument in stage 5. This study sought to achieve values of % agreement up to 0.9^21^, I-CVI of 0.78 or higher, and S-CVI/Ave of 0.90 or higher.^24^

Furthermore, the COSMIN (COnsensus-based Standards for the selection of health Measurement INstruments) was employed. This checklist includes 12 design requirements, evaluated in four categories (from very good up to inadequate), proposed to assess the quality of the translation process of “Patient report outcome measures”.^25^

## RESULTS

In stage 1, there were different translations for the following common use terms: “computing”, “screen”, “step” and “to the highest applicable”, because the translators used terms with similar meanings in Brazilian Portuguese. Only two medical terms received different translations: “patent ductus” and “exchange transfusion for hyperbilirubinemia”. Despite the translation of most of the medical terms being equal between the translators, the *naïve* translator informed that he did not know the meaning of the terms.

In stage 2, in the T3 version, the translators opted for the clearest and most grammatically appropriate terms in Brazilian Portuguese for choice of common use terms, and when there was a different translation of some medical terms, the *naïve* translator opted for the terms utilized by the nurse translator. In addition, they preferred to replace the abbreviations with the corresponding terms. The main researcher made a record of the decisions without interfering with the choice of terms.

In stage 3, a few terms of the T3 were back translated differently by the translators: “calculating/measuring”, “show below/presented” “seizures/convulsions” and the title of the instrument: “Neonatal Assessment Scale/Neonatal Medical Index”. Except for this last term, the others can be considered synonymous.

In stage 4, the EC first round, insufficient values of % agreement and CVI of some items related to the instructions for application of the instrument were attained, and an item that described the population of the original study which was considered unnecessary to NMI classification was excluded. Besides that, the experts considered inappropriate the translation into Brazilian Portuguese of the terms “exchange transfusion”, “respiratory distress syndrome”, “patent ductus” and “major surgery”. They also opted for the replacement of the drugs names specified in the original instrument by the expression “that required medications”.

In the EC second round, the desired values for the % agreement and CVI (Table 1) were reached and the pre-test version was prepared to be evaluated by the health professional participants in the prefinal version stage. In addition, the EC defined that the title of the Brazilian version of the NMI would be “*Índice Clínico Neonatal* (NMI-Br)”.

**Table 1:** Percentage of agreement, item and average content validity index for the experts committee of the *Índice Clínico Neonatal* (NMI-Br), Brazilian version of the Neonatal Medical Index.^3^. Florianópolis, Santa Catarina, Brazil, 2022.

	Round 1	Round 2
Percentage agreement		
Domains		
Clarity	60.0	91.0
Semantic equivalence	88.3	97.0
Idiomatic equivalence	83.0	100
Conceptual equivalence	80.0	100
Operational equivalence	95.0	100
Compatibility between versions	90.0	100
Entire instrument	84.4	97.0
I-CVI clarity		
Item		
1	0.80	0.90
2	1.00	1.00
3	1.00	1.00
4	1.00	1.00
5	1.00	0.90
6	0.80	1.00
7	0.60	1.00
8	0.90	1.00
9	1.00	1.00
10	1.00	1.00
11	0.66	Excluded*
S-CVI/Ave clarity	0.73	0.98
I-CVI representativeness		
1	1.00	1.00
2	1.00	1.00
3	1.00	1.00
4	1.00	1.00
5	1.00	1.00
6	1.00	1.00
7	0.90	1.00
8	1.00	1.00
9	1.00	1.00
10	1.00	1.00
11	0.77	Excluded*
S-CVI/Ave representativeness	0.88	1.00

I-CVI: item content validity index; S-CVI/Ave: average content validity index; I-CVI: item content validity index; S-CVI/Ave: average content validity index.

*Item 11 was excluded, because it was not considered clear or representative to the *Índice Clínico Neonatal* (NMI-Br), Brazilian version of the “Neonatal Medical Index”

In stage 5, the prefinal version was tested with 94 health professionals, of whom 75 were physical therapists (80%), 9 nurses (10%), 5 doctors (5%), 4 speech therapists (4%) and 1 was occupational therapist (1%), from different geographic regions of Brazil (38% in the South, 28% in the Northeast, 21% Southeast, 9% Central-West, and 4% North). All professionals had experience in NICU working: <6 months (8%), 6 months–1 year (10%), 1–5 years (32%), and >5 years (50%). They scored the clarity of the prefinal version that was organized into eight items to contemplate the layout of the original version. In this evaluation, one item attained I-CVI=0.86 and all the others achieved I-CVI>0.96 and the S-CVI/Ave=0.99. The values are presented in Table 2.

**Table 2:** Item and average content validity index for the participants of the test of the prefinal version of the *Índice Clínico Neonatal* (NMI-Br), Brazilian version of the Neonatal Medical Index.^3^ Florianópolis, Santa Catarina, Brazil, 2022.

Item	I-CVI clarity
1	0.97
2	1.00
3	0.97
4	0.86
5	0.98
6	0.99
7	0.96
8	0.99
S-CVI/Ave	0.99

I-CVI: item content validity index; S-CVI/Ave: average content validity index.

**Figure 2:**
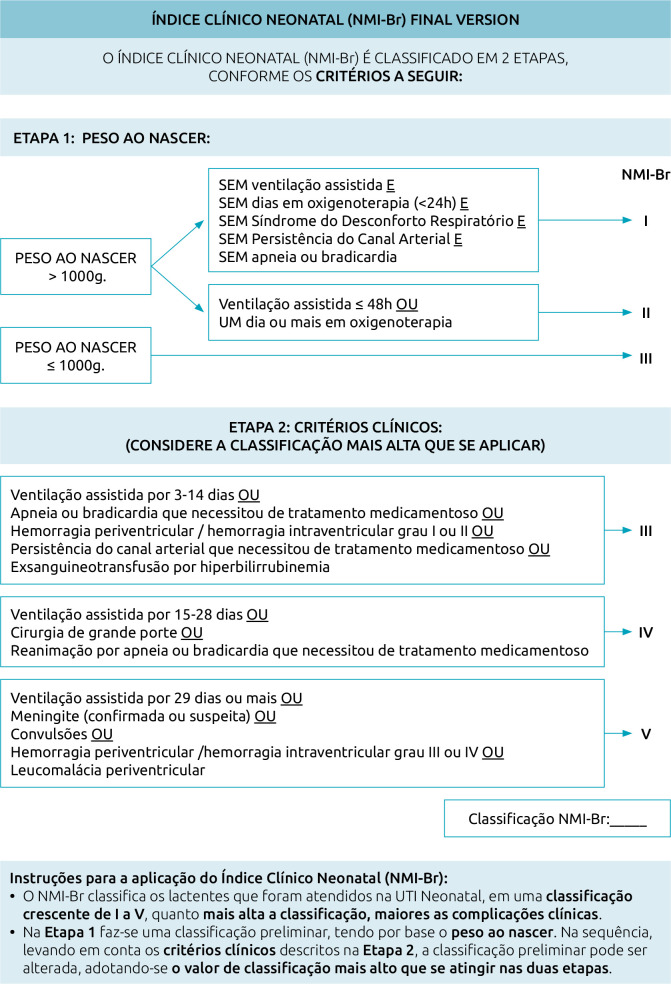
Final version of *Índice Clínico Neonatal* (NMI-Br), Brazilian version of the Neonatal Medical Index.^3^ Florianópolis, Santa Catarina, Brazil, 2022.

During the CCA, Dr. Stevenson and Dr. Feldman reviewed the back-translations synthesis and the prefinal version and authorized to continue the prefinal test. When the final version of the NMI-Br was ready, they were consulted again, and Dr. Feldman suggested some modifications in the order of the items and scores of the NMI-Br. The researchers evaluated these suggestions and considered that the classification process became easier to perform, and adopted them in the final version of the instrument (Figure 2).

Considering the 12 requirements for the CCA design proposed by COSMIN, this study achieved the highest classification (very good) for all of them.

## DISCUSSION

This CCA study of the NMI followed all stages and recommendations proposed by Beaton et al.^20^ It has achieved semantic, idiomatic, experimental and conceptual equivalence, and obtained the highest evaluation category for all requirements according to COSMIN guidelines.^25^

A contributing factor that may have enhanced the excellence of the translation process was the diversity within the EC. The clinical experience served as a selection criterion for EC members.^22^ To encompass a wide range of perspectives on the instrument’s content, individuals with varying professional specialties and different lengths of experience in the NICU were chosen. Furthermore, these professionals hailed from diverse geographic regions across Brazil to increase the likelihood of identifying colloquial terms, considering the country’s rich cultural diversity.^22^ The same criteria were applied when selecting participants for the prefinal version evaluation, ensuring that the items and instructions in the adapted version were clear.^21,22,25^

Another important aspect was the inclusion of a preterm infant’s mother in the EC, aiming to incorporate the assessment and opinion of a representative from the population evaluated by the NMI during its CCA and to enhance knowledge translation to this specific group.^26^

The major adaptations addressed during CCA involved:

1. Replacing the drug names specified in the original instrument with a broader expression and
2. Modifying the instrument application.

The first adaptation was necessary because the drugs described in the original version were not commonly prescribed in Brazil, likely reflecting changes in drug treatment since the instrument publication in the 1990s. The second modification was made in response to feedback from the first round of EC evaluation (I-CVI=0.6). Additionally, the last item, which indicated that some data were unavailable for the original study sample, received low CVI values in the first round of the EC evaluation (clarity I-CVI=0.66 and representativeness I-CVI=0.77), and was subsequently discarded as it was deemed unnecessary for the instrument use.

Following these adaptations, the NMI achieved a high expert % agreement value (98%), demonstrating its equivalence with the original instrument.^21,22^ It obtained maximum values for representativeness in both the I-CVI and S-CVI/Ave (1.00), indicating total agreement and being free from agreement due to chance factors.^24^ Additionally, it received high values for clarity in the I-CVI (all >0.97) and S-CVI/Ave (0.98) for the EC, as well as for the prefinal version test participants (all I-CVI>0.86 and S-CVI/Ave=0.99). The above-mentioned modifications align with the specialized literature,^21,22,24^ which emphasizes the use of I-CVI to guide EC decisions, along with the importance of obtaining I-CVI values of 0.78 or higher and S-CVI/Ave values of 0.9 or higher to be considered as having excellent content validity.^22,23,24^ These findings attest to the equivalence of the NMI-Br with the original instrument and support its application in pediatric and neonatal clinical practice.

In a clinical and research context, premature infants are presently categorized based on gestational age or birth weight.^1^ However, relying solely on these criteria does not adequately capture the complications experienced during NICU stay, which can significantly impact an infant’s subsequent health condition, especially for very and extremely preterm infants.^27,28^ In light of this, the NMI-Br provides a valuable option for classifying preterm infants in clinical practice. It not only incorporates the infant’s hospitalization health history but also offers the advantage of using data readily available in the hospital discharge report, thereby facilitating its practical application.

Since its creation, the NMI has served as a classification system in various research studies, including those investigating infant development,^4,6,7^ infant developmental prognostic factors,^6^ feeding routines in the NICU,^8^ early intervention programs^10,11^, and parenting stress.^9^ This exemplifies the diverse possibilities of NMI-Br utilization in the research context. However, it is noteworthy that only one of these studies was developed in Brazil^6^, and the NMI is still employed in the English version. With the availability of the adapted version in Brazil, it can now be applied to different research studies focused on preterm infants. Early care and support provision for infants with developmental disabilities in low-income settings are frequently lacking, despite the potential to improve infant and family outcomes.^29^ Considering the influence of social inequalities on access to quality early intervention programs for Brazilian premature infants and the current “wait and see” approach utilized by health services for outpatient follow-up, which involves detecting deviations in neurodevelopment and then referring the infant to early intervention services, proposing the CCA, translation and assess the content validity of the NMI-Br can help address this regulatory gap.

The NMI was developed in the 1990s, and its predictive validity for cognitive and motor development at 3 years of age,^3^ as well as its influence on neuromotor function and school performance at 7 years of age^4^ were primarily demonstrated in infants weighing <1500g. However, in the last three decades, significant advancements in neonatal intensive care, such as surfactant therapy,^30^ antenatal^31^ and postnatal corticosteroid treatment have emerged, leading to reduced mortality rates and potential changes in outcomes for preterm infants. Moreover, conditions such as bronchopulmonary dysplasia, necrotizing enterocolitis, and sepsis^27^ are now well-established as significant contributors to adverse outcomes in premature infants. However, these factors were not directly described in the original NMI version, nor were they addressed in the Brazilian CCA. Hence, it becomes crucial to evaluate whether the NMI continues to serve as a valid predictor of outcomes, considering these advancements in NICU treatment. Additionally, besides the need for updating the instrument, it is crucial to take into account that the now NMI-Br proposed in Brazil operates within a cultural context that is significantly different from the North American one. Therefore, it is imperative to verify whether the instrument remains a valid predictor of outcomes for the Brazilian population of preterm infants. The “*Índice Clínico Neonatal-NMI-Br*” is the Brazilian version of the “Neonatal Medical Index”, which underwent a successful translation and CCA process, adhering to an internationally recognized guideline. The NMI-Br achieved excellent content validity measure values and met the highest classification of all design requirements proposed by the COSMIN checklist for the translation process.

The NMI-Br classifies preterm infants based on their NICU history and proves to be a valuable tool in clinical practice in Brazil, particularly in the follow-up strategies for premature infants discharged from the NICU and research studies.

## Data Availability

The database that originated the article is available with the corresponding author.
